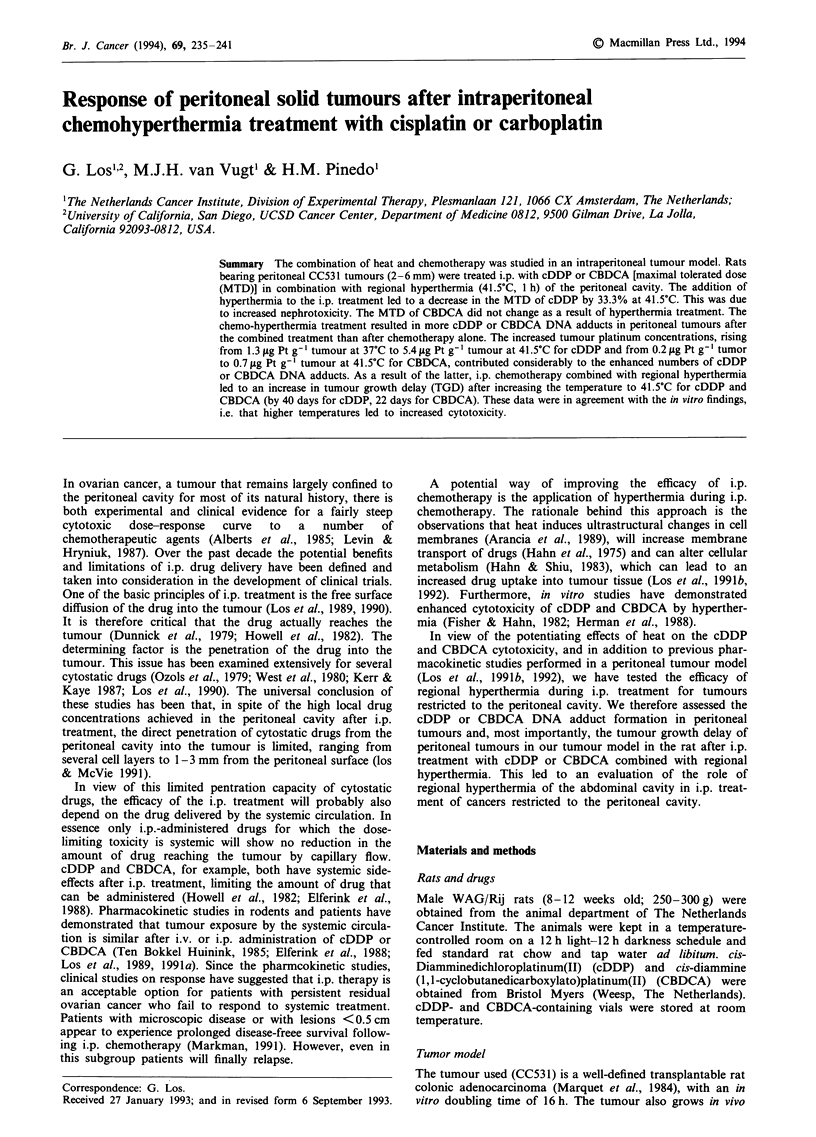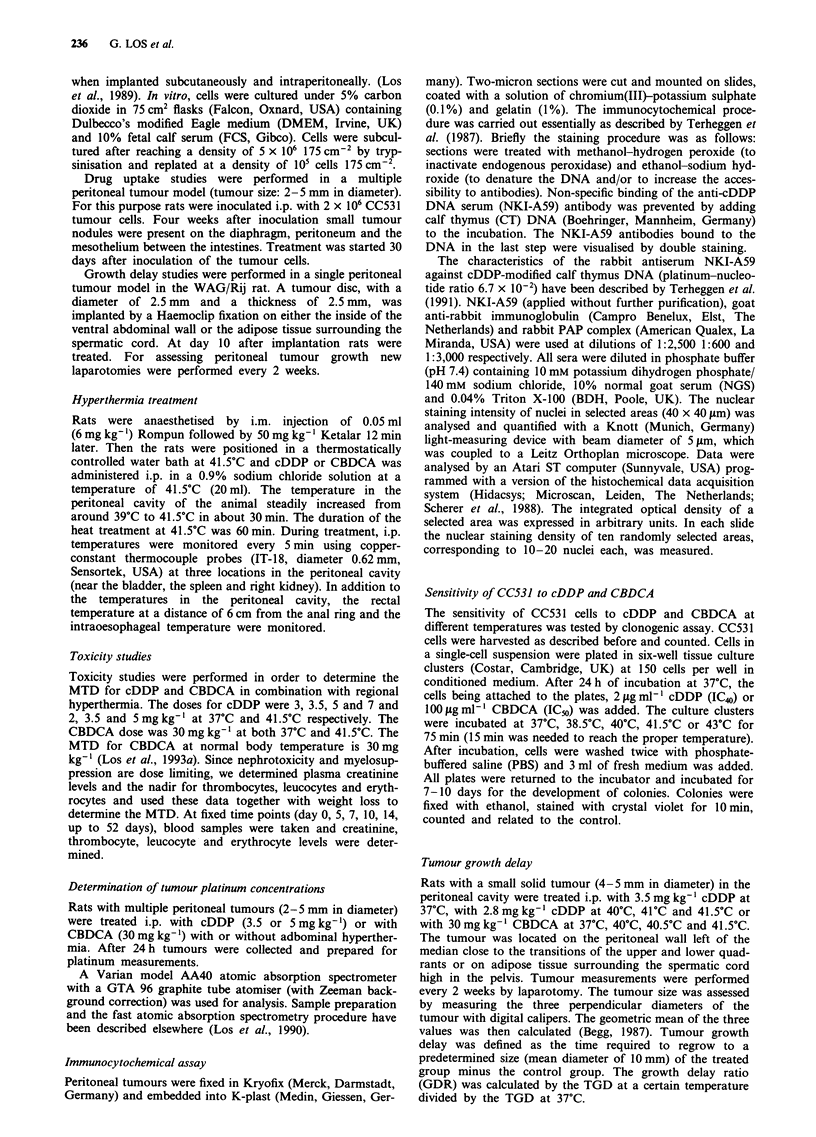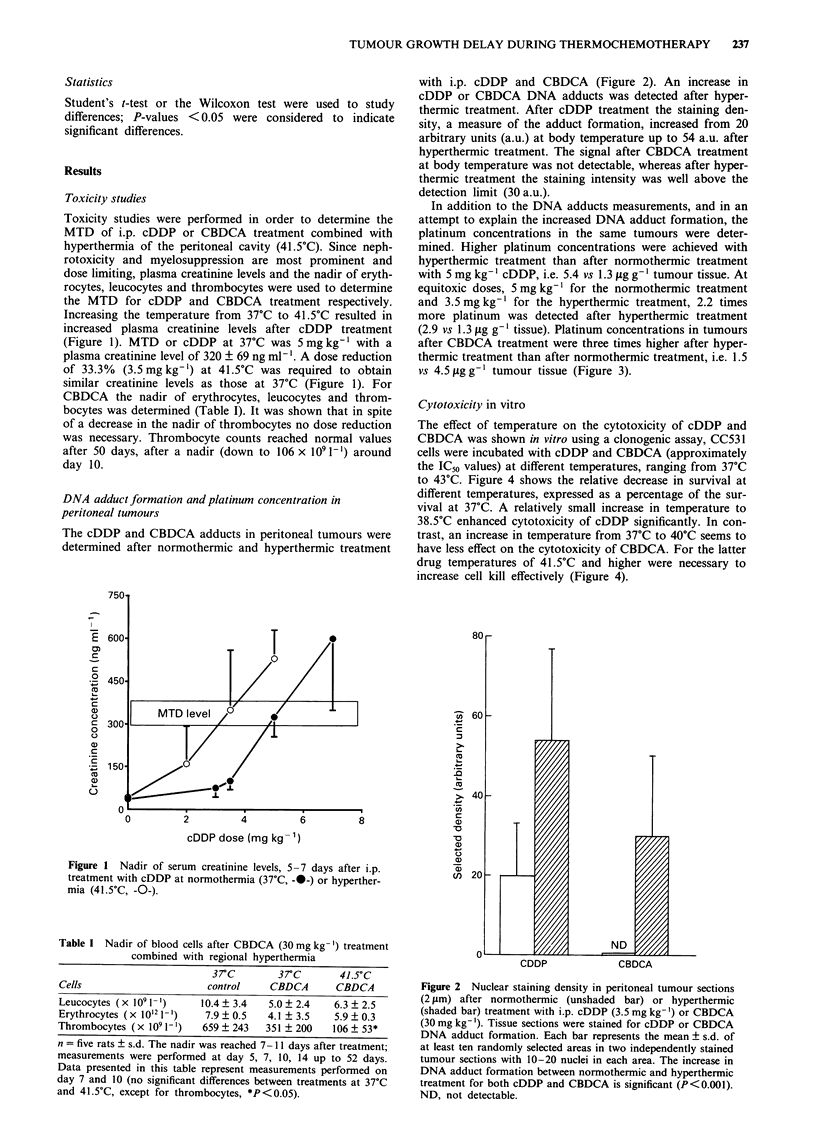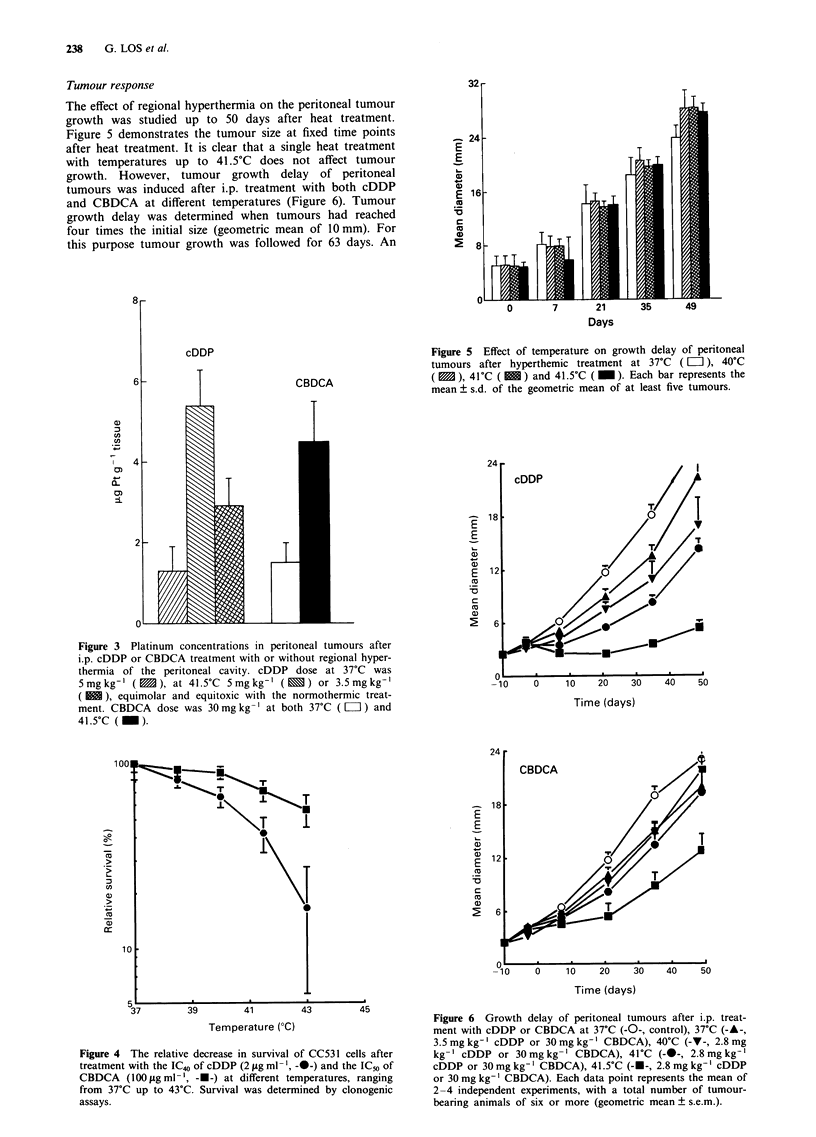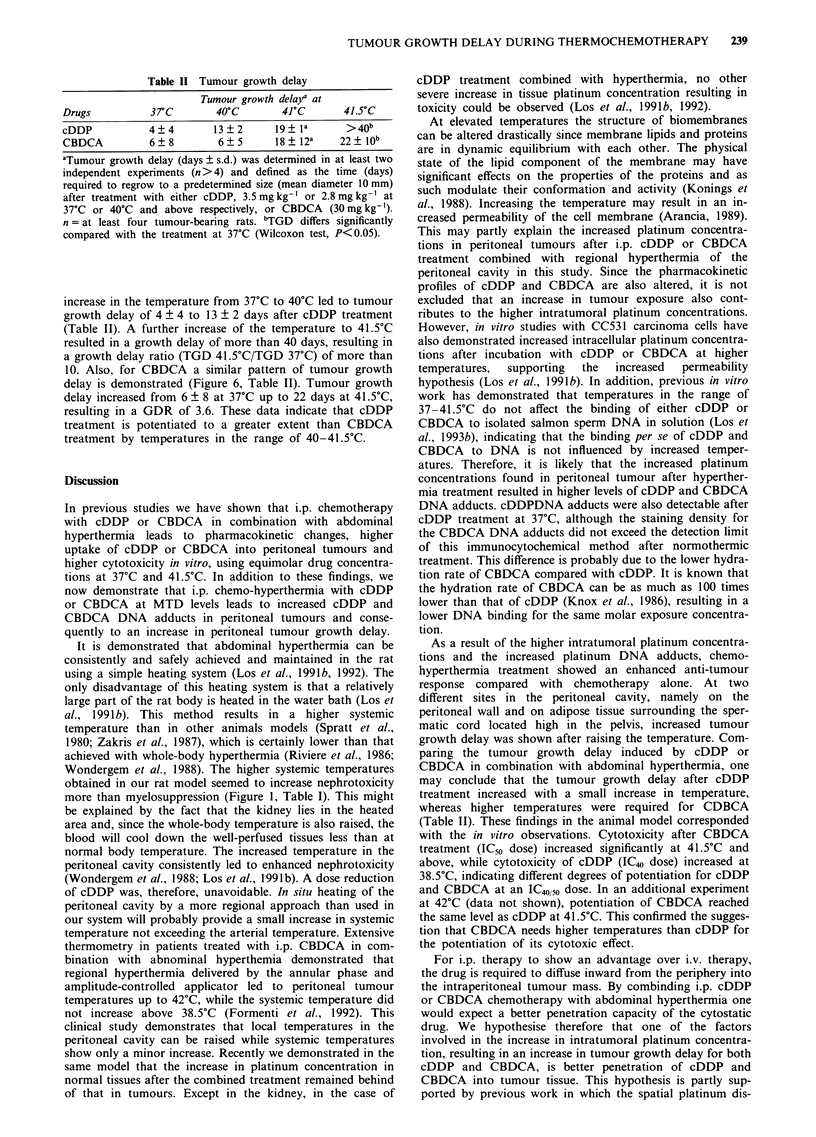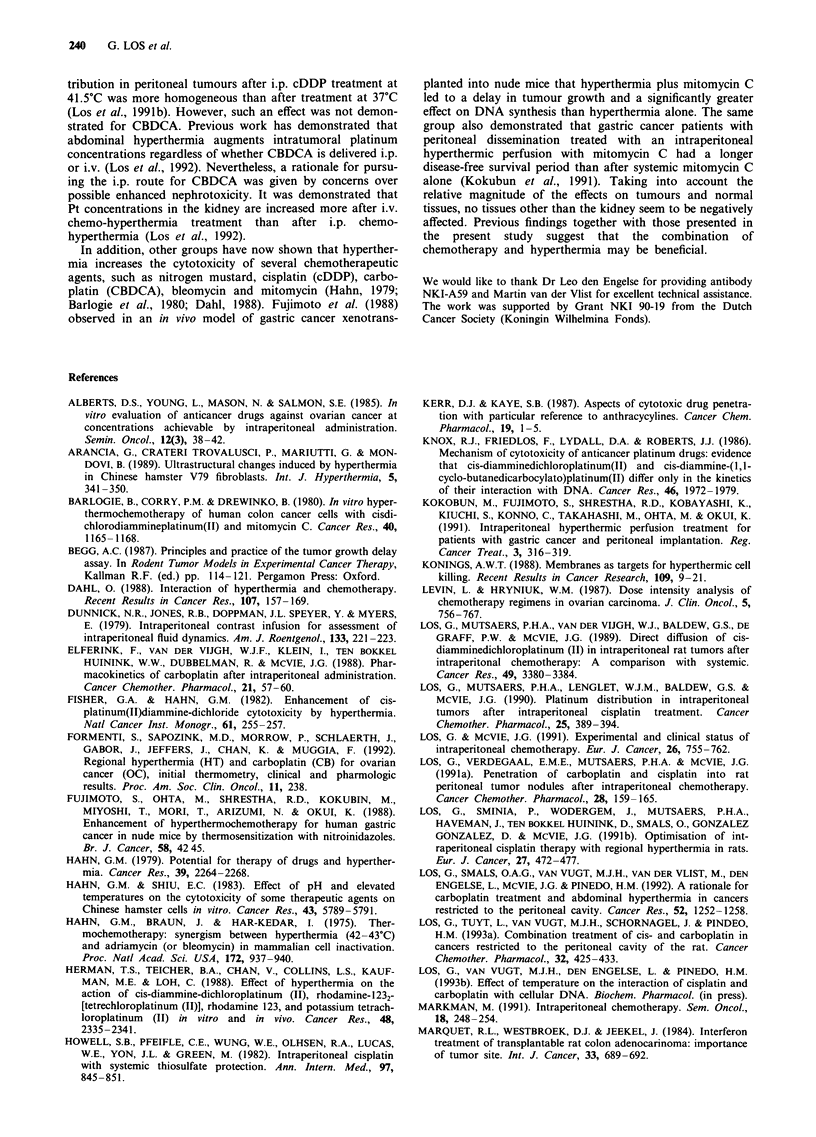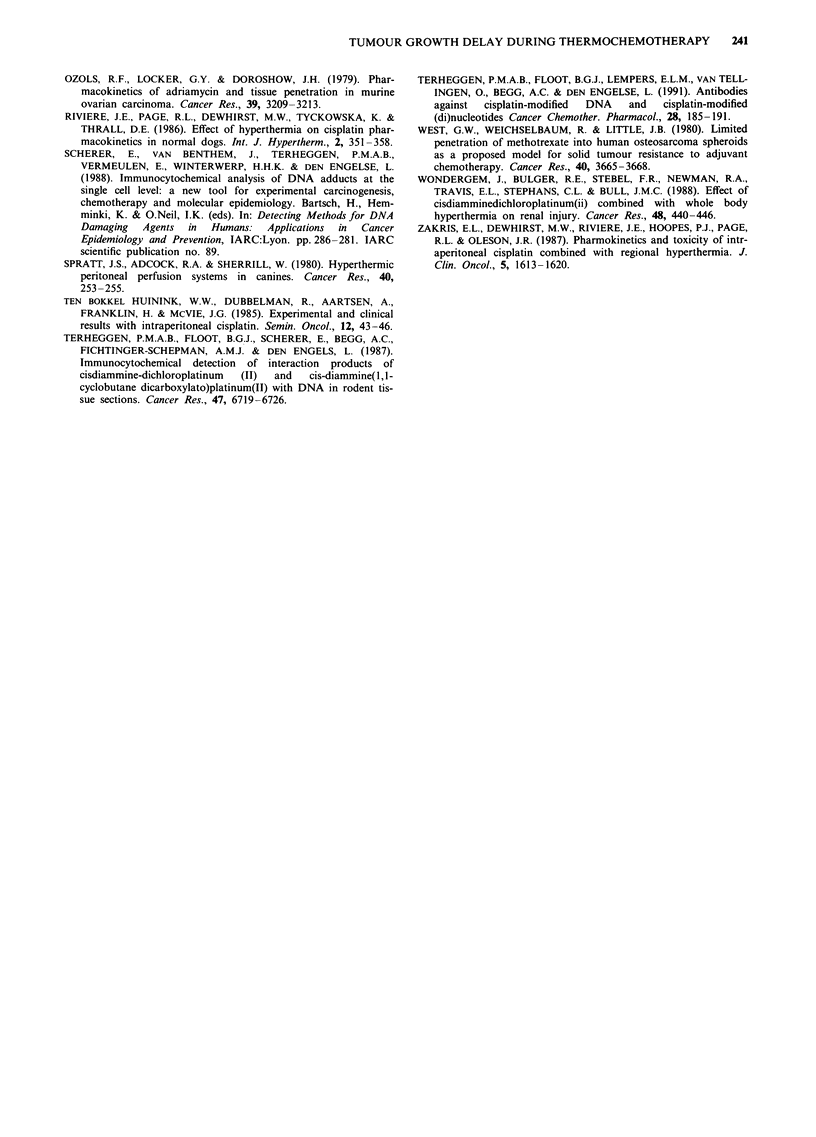# Response of peritoneal solid tumours after intraperitoneal chemohyperthermia treatment with cisplatin or carboplatin.

**DOI:** 10.1038/bjc.1994.45

**Published:** 1994-02

**Authors:** G. Los, M. J. van Vugt, H. M. Pinedo

**Affiliations:** The Netherlands Cancer Institute, Division of Experimental Therapy, Amsterdam.

## Abstract

The combination of heat and chemotherapy was studied in an intraperitoneal tumour model. Rats bearing peritoneal CC531 tumours (2-6 mm) were treated i.p. with cDDP or CBDCA [maximal tolerated dose (MTD)] in combination with regional hyperthermia (41.5 degrees C, 1 h) of the peritoneal cavity. The addition of hyperthermia to the i.p. treatment led to a decrease in the MTD of cDDP by 33.3% at 41.5 degrees C. This was due to increased nephrotoxicity. The MTD of CBDCA did not change as a result of hyperthermia treatment. The chemo-hyperthermia treatment resulted in more cDDP or CBDCA DNA adducts in peritoneal tumours after the combined treatment than after chemotherapy alone. The increased tumour platinum concentrations, rising from 1.3 micrograms Pt g-1 tumour at 37 degrees C to 5.4 micrograms Pt g-1 tumour at 41.5 degrees C for cDDP and from 0.2 microgram Pt g-1 tumor to 0.7 microgram Pt g-1 tumour at 41.5 degrees C for CBDCA, contributed considerably to the enhanced numbers of cDDP or CBDCA DNA adducts. As a result of the latter, i.p. chemotherapy combined with regional hyperthermia led to an increase in tumour growth delay (TGD) after increasing the temperature to 41.5 degrees C for cDDP and CBDCA (by 40 days for cDDP, 22 days for CBDCA). These data were in agreement with the in vitro findings, i.e. that higher temperatures led to increased cytotoxicity.


Br. J. Cancer (1994), 69, 235–241 © Macmillan Press Ltd., 1994

### Response of peritoneal solid tumours after intraperitoneal chemohyperthermia treatment with cisplatin or carboplatin

G. Los[1,2], M.J.H. van Vugt[1] & H.M. Pinedo[1]

[1]*The Netherlands Cancer Institute, Division of Experimental Therapy, Plesmanlaan 121, 1066 CX Amsterdam, The Netherlands;* [2]*University of California, San Diego, UCSD Cancer Center, Department of Medicine 0812, 9500 Gilman Drive, La Jolla, California 92093-0812, USA.*

**Summary** The combination of heat and chemotherapy was studied in an intraperitoneal tumour model. Rats bearing peritoneal CC531 tumours (2–6 mm) were treated i.p. with cDDP or CBDCA [maximal tolerated dose (MTD)] in combination with regional hyperthermia (41.5°C, 1 h) of the peritoneal cavity. The addition of hyperthermia to the i.p. treatment led to a decrease in the MTD of cDDP by 33.3% at 41.5°C. This was due to increased nephrotoxicity. The MTD of CBDCA did not change as a result of hyperthermia treatment. The chemo-hyperthermia treatment resulted in more cDDP or CBDCA DNA adducts in peritoneal tumours after the combined treatment than after chemotherapy alone. The increased tumour platinum concentrations, rising from 1.3 μg Pt $g^{-1}$ tumour at 37°C to 5.4 μg Pt $g^{-1}$ tumour at 41.5°C for cDDP and from 0.2 μg Pt $g^{-1}$ tumor to 0.7 μg Pt $g^{-1}$ tumour at 41.5°C for CBDCA, contributed considerably to the enhanced numbers of cDDP or CBDCA DNA adducts. As a result of the latter, i.p. chemotherapy combined with regional hyperthermia led to an increase in tumour growth delay (TGD) after increasing the temperature to 41.5°C for cDDP and CBDCA (by 40 days for cDDP, 22 days for CBDCA). These data were in agreement with the *in vitro* findings, i.e. that higher temperatures led to increased cytotoxicity.

In ovarian cancer, a tumour that remains largely confined to the peritoneal cavity for most of its natural history, there is both experimental and clinical evidence for a fairly steep cytotoxic dose–response curve to a number of chemotherapeutic agents (Alberts *et al.*, 1985; Levin & Hryniuk, 1987). Over the past decade the potential benefits and limitations of i.p. drug delivery have been defined and taken into consideration in the development of clinical trials. One of the basic principles of i.p. treatment is the free surface diffusion of the drug into the tumour (Los *et al.*, 1989, 1990). It is therefore critical that the drug actually reaches the tumour (Dunnick *et al.*, 1979; Howell *et al.*, 1982). The determining factor is the penetration of the drug into the tumour. This issue has been examined extensively for several cytostatic drugs (Ozols *et al.*, 1979; West *et al.*, 1980; Kerr & Kaye 1987; Los *et al.*, 1990). The universal conclusion of these studies has been that, in spite of the high local drug concentrations achieved in the peritoneal cavity after i.p. treatment, the direct penetration of cytostatic drugs from the peritoneal cavity into the tumour is limited, ranging from several cell layers to 1–3 mm from the peritoneal surface (los & McVie 1991).

In view of this limited pentration capacity of cytostatic drugs, the efficacy of the i.p. treatment will probably also depend on the drug delivered by the systemic circulation. In essence only i.p.-administered drugs for which the dose-limiting toxicity is systemic will show no reduction in the amount of drug reaching the tumour by capillary flow. cDDP and CBDCA, for example, both have systemic side-effects after i.p. treatment, limiting the amount of drug that can be administered (Howell *et al.*, 1982; Elferink *et al.*, 1988). Pharmacokinetic studies in rodents and patients have demonstrated that tumour exposure by the systemic circulation is similar after i.v. or i.p. administration of cDDP or CBDCA (Ten Bokkel Huinink, 1985; Elferink *et al.*, 1988; Los *et al.*, 1989, 1991*a*). Since the pharmcokinetic studies, clinical studies on response have suggested that i.p. therapy is an acceptable option for patients with persistent residual ovarian cancer who fail to respond to systemic treatment. Patients with microscopic disease or with lesions <0.5 cm appear to experience prolonged disease-freee survival following i.p. chemotherapy (Markman, 1991). However, even in this subgroup patients will finally relapse.

Correspondence: G. Los.
Received 27 January 1993; and in revised form 6 September 1993.

A potential way of improving the efficacy of i.p. chemotherapy is the application of hyperthermia during i.p. chemotherapy. The rationale behind this approach is the observations that heat induces ultrastructural changes in cell membranes (Arancia *et al.*, 1989), will increase membrane transport of drugs (Hahn *et al.*, 1975) and can alter cellular metabolism (Hahn & Shiu, 1983), which can lead to an increased drug uptake into tumour tissue (Los *et al.*, 1991*b*, 1992). Furthermore, *in vitro* studies have demonstrated enhanced cytotoxicity of cDDP and CBDCA by hyperthermia (Fisher & Hahn, 1982; Herman *et al.*, 1988).

In view of the potentiating effects of heat on the cDDP and CBDCA cytotoxicity, and in addition to previous pharmacokinetic studies performed in a peritoneal tumour model (Los *et al.*, 1991*b*, 1992), we have tested the efficacy of regional hyperthermia during i.p. treatment for tumours restricted to the peritoneal cavity. We therefore assessed the cDDP or CBDCA DNA adduct formation in peritoneal tumours and, most importantly, the tumour growth delay of peritoneal tumours in our tumour model in the rat after i.p. treatment with cDDP or CBDCA combined with regional hyperthermia. This led to an evaluation of the role of regional hyperthermia of the abdominal cavity in i.p. treatment of cancers restricted to the peritoneal cavity.

### Materials and methods

### *Rats and drugs*

Male WAG/Rij rats (8–12 weeks old; 250–300 g) were obtained from the animal department of The Netherlands Cancer Institute. The animals were kept in a temperature-controlled room on a 12 h light–12 h darkness schedule and fed standard rat chow and tap water *ad libitum*. *cis*-Diamminedichloroplatinum(II) (cDDP) and *cis*-diammine (1,1-cyclobutanedicarboxylato)platinum(II) (CBDCA) were obtained from Bristol Myers (Weesp, The Netherlands). cDDP- and CBDCA-containing vials were stored at room temperature.

### *Tumor model*

The tumour used (CC531) is a well-defined transplantable rat colonic adenocarcinoma (Marquet *et al.*, 1984), with an *in vitro* doubling time of 16 h. The tumour also grows *in vivo*

when implanted subcutaneously and intraperitoneally. (Los *et al.*, 1989). *In vitro*, cells were cultured under 5% carbon dioxide in 75 $cm^2$ flasks (Falcon, Oxnard, USA) containing Dulbecco's modified Eagle medium (DMEM, Irvine, UK) and 10% fetal calf serum (FCS, Gibco). Cells were subcultured after reaching a density of $5 \times 10^6$ 175 $cm^{-2}$ by trypsinisation and replated at a density of $10^5$ cells 175 $cm^{-2}$.

Drug uptake studies were performed in a multiple peritoneal tumour model (tumour size: 2–5 mm in diameter). For this purpose rats were inoculated i.p. with $2 \times 10^6$ CC531 tumour cells. Four weeks after inoculation small tumour nodules were present on the diaphragm, peritoneum and the mesothelium between the intestines. Treatment was started 30 days after inoculation of the tumour cells.

Growth delay studies were performed in a single peritoneal tumour model in the WAG/Rij rat. A tumour disc, with a diameter of 2.5 mm and a thickness of 2.5 mm, was implanted by a Haemoclip fixation on either the inside of the ventral abdominal wall or the adipose tissue surrounding the spermatic cord. At day 10 after implantation rats were treated. For assessing peritoneal tumour growth new laparotomies were performed every 2 weeks.

*Hyperthermia treatment*

Rats were anaesthetised by i.m. injection of 0.05 ml (6 mg $kg^{-1}$) Rompun followed by 50 mg $kg^{-1}$ Ketalar 12 min later. Then the rats were positioned in a thermostatically controlled water bath at 41.5°C and cDDP or CBDCA was administered i.p. in a 0.9% sodium chloride solution at a temperature of 41.5°C (20 ml). The temperature in the peritoneal cavity of the animal steadily increased from around 39°C to 41.5°C in about 30 min. The duration of the heat treatment at 41.5°C was 60 min. During treatment, i.p. temperatures were monitored every 5 min using copper-constant thermocouple probes (IT-18, diameter 0.62 mm, Sensortek, USA) at three locations in the peritoneal cavity (near the bladder, the spleen and right kidney). In addition to the temperatures in the peritoneal cavity, the rectal temperature at a distance of 6 cm from the anal ring and the intraoesophageal temperature were monitored.

*Toxicity studies*

Toxicity studies were performed in order to determine the MTD for cDDP and CBDCA in combination with regional hyperthermia. The doses for cDDP were 3, 3.5, 5 and 7 and 2, 3.5 and 5 mg $kg^{-1}$ at 37°C and 41.5°C respectively. The CBDCA dose was 30 mg $kg^{-1}$ at both 37°C and 41.5°C. The MTD for CBDCA at normal body temperature is 30 mg $kg^{-1}$ (Los *et al.*, 1993*a*). Since nephrotoxicity and myelosuppression are dose limiting, we determined plasma creatinine levels and the nadir for thrombocytes, leucocytes and erythrocytes and used these data together with weight loss to determine the MTD. At fixed time points (day 0, 5, 7, 10, 14, up to 52 days), blood samples were taken and creatinine, thrombocyte, leucocyte and erythrocyte levels were determined.

*Determination of tumour platinum concentrations*

Rats with multiple peritoneal tumours (2–5 mm in diameter) were treated i.p. with cDDP (3.5 or 5 mg $kg^{-1}$) or with CBDCA (30 mg $kg^{-1}$) with or without adbominal hyperthermia. After 24 h tumours were collected and prepared for platinum measurements.

A Varian model AA40 atomic absorption spectrometer with a GTA 96 graphite tube atomiser (with Zeeman background correction) was used for analysis. Sample preparation and the fast atomic absorption spectrometry procedure have been described elsewhere (Los *et al.*, 1990).

*Immunocytochemical assay*

Peritoneal tumours were fixed in Kryofix (Merck, Darmstadt, Germany) and embedded into K-plast (Medin, Giessen, Germany). Two-micron sections were cut and mounted on slides, coated with a solution of chromium(III)–potassium sulphate (0.1%) and gelatin (1%). The immunocytochemical procedure was carried out essentially as described by Terheggen *et al.* (1987). Briefly the staining procedure was as follows: sections were treated with methanol–hydrogen peroxide (to inactivate endogenous peroxidase) and ethanol–sodium hydroxide (to denature the DNA and/or to increase the accessibility to antibodies). Non-specific binding of the anti-cDDP DNA serum (NKI-A59) antibody was prevented by adding calf thymus (CT) DNA (Boehringer, Mannheim, Germany) to the incubation. The NKI-A59 antibodies bound to the DNA in the last step were visualised by double staining.

The characteristics of the rabbit antiserum NKI-A59 against cDDP-modified calf thymus DNA (platinum–nucleotide ratio $6.7 \times 10^{-2}$) have been described by Terheggen *et al.* (1991). NKI-A59 (applied without further purification), goat anti-rabbit immunoglobulin (Campro Benelux, Elst, The Netherlands) and rabbit PAP complex (American Qualex, La Miranda, USA) were used at dilutions of 1:2,500 1:600 and 1:3,000 respectively. All sera were diluted in phosphate buffer (pH 7.4) containing 10 mM potassium dihydrogen phosphate/ 140 mM sodium chloride, 10% normal goat serum (NGS) and 0.04% Triton X-100 (BDH, Poole, UK). The nuclear staining intensity of nuclei in selected areas (40 × 40 μm) was analysed and quantified with a Knott (Munich, Germany) light-measuring device with beam diameter of 5 μm, which was coupled to a Leitz Orthoplan microscope. Data were analysed by an Atari ST computer (Sunnyvale, USA) programmed with a version of the histochemical data acquisition system (Hidacsys; Microscan, Leiden, The Netherlands; Scherer *et al.*, 1988). The integrated optical density of a selected area was expressed in arbitrary units. In each slide the nuclear staining density of ten randomly selected areas, corresponding to 10–20 nuclei each, was measured.

*Sensitivity of CC531 to cDDP and CBDCA*

The sensitivity of CC531 cells to cDDP and CBDCA at different temperatures was tested by clonogenic assay. CC531 cells were harvested as described before and counted. Cells in a single-cell suspension were plated in six-well tissue culture clusters (Costar, Cambridge, UK) at 150 cells per well in conditioned medium. After 24 h of incubation at 37°C, the cells being attached to the plates, 2 μg $ml^{-1}$ cDDP ($IC_{40}$) or 100 μg $ml^{-1}$ CBDCA ($IC_{50}$) was added. The culture clusters were incubated at 37°C, 38.5°C, 40°C, 41.5°C or 43°C for 75 min (15 min was needed to reach the proper temperature). After incubation, cells were washed twice with phosphate-buffered saline (PBS) and 3 ml of fresh medium was added. All plates were returned to the incubator and incubated for 7–10 days for the development of colonies. Colonies were fixed with ethanol, stained with crystal violet for 10 min, counted and related to the control.

*Tumour growth delay*

Rats with a small solid tumour (4–5 mm in diameter) in the peritoneal cavity were treated i.p. with 3.5 mg $kg^{-1}$ cDDP at 37°C, with 2.8 mg $kg^{-1}$ cDDP at 40°C, 41°C and 41.5°C or with 30 mg $kg^{-1}$ CBDCA at 37°C, 40°C, 40.5°C and 41.5°C. The tumour was located on the peritoneal wall left of the median close to the transitions of the upper and lower quadrants or on adipose tissue surrounding the spermatic cord high in the pelvis. Tumour measurements were performed every 2 weeks by laparotomy. The tumour size was assessed by measuring the three perpendicular diameters of the tumour with digital calipers. The geometric mean of the three values was then calculated (Begg, 1987). Tumour growth delay was defined as the time required to regrow to a predetermined size (mean diameter of 10 mm) of the treated group minus the control group. The growth delay ratio (GDR) was calculated by the TGD at a certain temperature divided by the TGD at 37°C.

*Statistics*

Student's *t*-test or the Wilcoxon test were used to study differences; *P*-values $<0.05$ were considered to indicate significant differences.

### Results

*Toxicity studies*

Toxicity studies were performed in order to determine the MTD of i.p. cDDP or CBDCA treatment combined with hyperthermia of the peritoneal cavity (41.5°C). Since nephrotoxicity and myelosuppression are most prominent and dose limiting, plasma creatinine levels and the nadir of erythrocytes, leucocytes and thrombocytes were used to determine the MTD for cDDP and CBDCA treatment respectively. Increasing the temperature from 37°C to 41.5°C resulted in increased plasma creatinine levels after cDDP treatment (Figure 1). MTD or cDDP at 37°C was 5 mg $kg^{-1}$ with a plasma creatinine level of $320 \pm 69$ ng $ml^{-1}$. A dose reduction of 33.3% (3.5 mg $kg^{-1}$) at 41.5°C was required to obtain similar creatinine levels as those at 37°C (Figure 1). For CBDCA the nadir of erythrocytes, leucocytes and thrombocytes was determined (Table I). It was shown that in spite of a decrease in the nadir of thrombocytes no dose reduction was necessary. Thrombocyte counts reached normal values after 50 days, after a nadir (down to $106 \times 10^9 l^{-1}$) around day 10.

*DNA adduct formation and platinum concentration in peritoneal tumours*

The cDDP and CBDCA adducts in peritoneal tumours were determined after normothermic and hyperthermic treatment with i.p. cDDP and CBDCA (Figure 2). An increase in cDDP or CBDCA DNA adducts was detected after hyperthermic treatment. After cDDP treatment the staining density, a measure of the adduct formation, increased from 20 arbitrary units (a.u.) at body temperature up to 54 a.u. after hyperthermic treatment. The signal after CBDCA treatment at body temperature was not detectable, whereas after hyperthermic treatment the staining intensity was well above the detection limit (30 a.u.).

In addition to the DNA adducts measurements, and in an attempt to explain the increased DNA adduct formation, the platinum concentrations in the same tumours were determined. Higher platinum concentrations were achieved with hyperthermic treatment than after normothermic treatment with 5 mg $kg^{-1}$ cDDP, i.e. 5.4 *vs* 1.3 $\mu g\ g^{-1}$ tumour tissue. At equitoxic doses, 5 mg $kg^{-1}$ for the normothermic treatment and 3.5 mg $kg^{-1}$ for the hyperthermic treatment, 2.2 times more platinum was detected after hyperthermic treatment (2.9 *vs* 1.3 $\mu g\ g^{-1}$ tissue). Platinum concentrations in tumours after CBDCA treatment were three times higher after hyperthermic treatment than after normothermic treatment, i.e. 1.5 *vs* 4.5 $\mu g\ g^{-1}$ tumour tissue (Figure 3).

*Cytotoxicity* in vitro

The effect of temperature on the cytotoxicity of cDDP and CBDCA was shown *in vitro* using a clonogenic assay, CC531 cells were incubated with cDDP and CBDCA (approximately the $IC_{50}$ values) at different temperatures, ranging from 37°C to 43°C. Figure 4 shows the relative decrease in survival at different temperatures, expressed as a percentage of the survival at 37°C. A relatively small increase in temperature to 38.5°C enhanced cytotoxicity of cDDP significantly. In contrast, an increase in temperature from 37°C to 40°C seems to have less effect on the cytotoxicity of CBDCA. For the latter drug temperatures of 41.5°C and higher were necessary to increase cell kill effectively (Figure 4).

**Figure 1** Nadir of serum creatinine levels, 5–7 days after i.p. treatment with cDDP at normothermia (37°C, -●-) or hyperthermia (41.5°C, -O-).

**Table I** Nadir of blood cells after CBDCA (30 mg $kg^{-1}$) treatment combined with regional hyperthermia

| *Cells* | *37°C control* | *37°C CBDCA* | *41.5°C CBDCA* |
|---|---|---|---|
| Leucocytes ($\times 10^9 l^{-1}$) | $10.4 \pm 3.4$ | $5.0 \pm 2.4$ | $6.3 \pm 2.5$ |
| Erythrocytes ($\times 10^{12} l^{-1}$) | $7.9 \pm 0.5$ | $4.1 \pm 3.5$ | $5.9 \pm 0.3$ |
| Thrombocytes ($\times 10^9 l^{-1}$) | $659 \pm 243$ | $351 \pm 200$ | $106 \pm 53$* |

$n$ = five rats ± s.d. The nadir was reached 7–11 days after treatment; measurements were performed at day 5, 7, 10, 14 up to 52 days. Data presented in this table represent measurements performed on day 7 and 10 (no significant differences between treatments at 37°C and 41.5°C, except for thrombocytes, *$P<0.05$).

**Figure 2** Nuclear staining density in peritoneal tumour sections (2 µm) after normothermic (unshaded bar) or hyperthermic (shaded bar) treatment with i.p. cDDP (3.5 mg $kg^{-1}$) or CBDCA (30 mg $kg^{-1}$). Tissue sections were stained for cDDP or CBDCA DNA adduct formation. Each bar represents the mean ± s.d. of at least ten randomly selected areas in two independently stained tumour sections with 10–20 nuclei in each area. The increase in DNA adduct formation between normothermic and hyperthermic treatment for both cDDP and CBDCA is significant ($P<0.001$). ND, not detectable.

*Tumour response*

The effect of regional hyperthermia on the peritoneal tumour growth was studied up to 50 days after heat treatment. Figure 5 demonstrates the tumour size at fixed time points after heat treatment. It is clear that a single heat treatment with temperatures up to 41.5°C does not affect tumour growth. However, tumour growth delay of peritoneal tumours was induced after i.p. treatment with both cDDP and CBDCA at different temperatures (Figure 6). Tumour growth delay was determined when tumours had reached four times the initial size (geometric mean of 10 mm). For this purpose tumour growth was followed for 63 days. An

**Figure 5** Effect of temperature on growth delay of peritoneal tumours after hyperthemic treatment at 37°C (□), 40°C (▨), 41°C (▩) and 41.5°C (■). Each bar represents the mean ± s.d. of the geometric mean of at least five tumours.

**Figure 3** Platinum concentrations in peritoneal tumours after i.p. cDDP or CBDCA treatment with or without regional hyperthermia of the peritoneal cavity. cDDP dose at 37°C was 5 mg kg$^{-1}$ (▨), at 41.5°C 5 mg kg$^{-1}$ (▧) or 3.5 mg kg$^{-1}$ (▩), equimolar and equitoxic with the normothermic treatment. CBDCA dose was 30 mg kg$^{-1}$ at both 37°C (□) and 41.5°C (■).

**Figure 6** Growth delay of peritoneal tumours after i.p. treatment with cDDP or CBDCA at 37°C (-O-, control), 37°C (-▲-, 3.5 mg kg$^{-1}$ cDDP or 30 mg kg$^{-1}$ CBDCA), 40°C (-▼-, 2.8 mg kg$^{-1}$ cDDP or 30 mg kg$^{-1}$ CBDCA), 41°C (-●-, 2.8 mg kg$^{-1}$ cDDP or 30 mg kg$^{-1}$ CBDCA), 41.5°C (-■-, 2.8 mg kg$^{-1}$ cDDP or 30 mg kg$^{-1}$ CBDCA). Each data point represents the mean of 2–4 independent experiments, with a total number of tumour-bearing animals of six or more (geometric mean ± s.e.m.).

**Figure 4** The relative decrease in survival of CC531 cells after treatment with the $IC_{40}$ of cDDP (2 μg ml$^{-1}$, -●-) and the $IC_{50}$ of CBDCA (100 μg ml$^{-1}$, -■-) at different temperatures, ranging from 37°C up to 43°C. Survival was determined by clonogenic assays.

**Table II** Tumour growth delay

| Drugs | 37°C | 40°C | 41°C | 41.5°C |
|---|---|---|---|---|
| | *Tumour growth delay*[a] *at* | | | |
| cDDP | $4 \pm 4$ | $13 \pm 2$ | $19 \pm 1$[a] | $>40$[b] |
| CBDCA | $6 \pm 8$ | $6 \pm 5$ | $18 \pm 12$[a] | $22 \pm 10$[b] |

[a]Tumour growth delay (days ± s.d.) was determined in at least two independent experiments ($n > 4$) and defined as the time (days) required to regrow to a predetermined size (mean diameter 10 mm) after treatment with either cDDP, 3.5 mg $kg^{-1}$ or 2.8 mg $kg^{-1}$ at 37°C or 40°C and above respectively, or CBDCA (30 mg $kg^{-1}$). $n$ = at least four tumour-bearing rats. [b]TGD differs significantly compared with the treatment at 37°C (Wilcoxon test, $P < 0.05$).

increase in the temperature from 37°C to 40°C led to tumour growth delay of 4 ± 4 to 13 ± 2 days after cDDP treatment (Table II). A further increase of the temperature to 41.5°C resulted in a growth delay of more than 40 days, resulting in a growth delay ratio (TGD 41.5°C/TGD 37°C) of more than 10. Also, for CBDCA a similar pattern of tumour growth delay is demonstrated (Figure 6, Table II). Tumour growth delay increased from 6 ± 8 at 37°C up to 22 days at 41.5°C, resulting in a GDR of 3.6. These data indicate that cDDP treatment is potentiated to a greater extent than CBDCA treatment by temperatures in the range of 40–41.5°C.

### Discussion

In previous studies we have shown that i.p. chemotherapy with cDDP or CBDCA in combination with abdominal hyperthermia leads to pharmacokinetic changes, higher uptake of cDDP or CBDCA into peritoneal tumours and higher cytotoxicity *in vitro*, using equimolar drug concentrations at 37°C and 41.5°C. In addition to these findings, we now demonstrate that i.p. chemo-hyperthermia with cDDP or CBDCA at MTD levels leads to increased cDDP and CBDCA DNA adducts in peritoneal tumours and consequently to an increase in peritoneal tumour growth delay.

It is demonstrated that abdominal hyperthermia can be consistently and safely achieved and maintained in the rat using a simple heating system (Los *et al.*, 1991*b*, 1992). The only disadvantage of this heating system is that a relatively large part of the rat body is heated in the water bath (Los *et al.*, 1991*b*). This method results in a higher systemic temperature than in other animals models (Spratt *et al.*, 1980; Zakris *et al.*, 1987), which is certainly lower than that achieved with whole-body hyperthermia (Riviere *et al.*, 1986; Wondergem *et al.*, 1988). The higher systemic temperatures obtained in our rat model seemed to increase nephrotoxicity more than myelosuppression (Figure 1, Table I). This might be explained by the fact that the kidney lies in the heated area and, since the whole-body temperature is also raised, the blood will cool down the well-perfused tissues less than at normal body temperature. The increased temperature in the peritoneal cavity consistently led to enhanced nephrotoxicity (Wondergem *et al.*, 1988; Los *et al.*, 1991b). A dose reduction of cDDP was, therefore, unavoidable. *In situ* heating of the peritoneal cavity by a more regional approach than used in our system will probably provide a small increase in systemic temperature not exceeding the arterial temperature. Extensive thermometry in patients treated with i.p. CBDCA in combination with abnominal hyperthemia demonstrated that regional hyperthermia delivered by the annular phase and amplitude-controlled applicator led to peritoneal tumour temperatures up to 42°C, while the systemic temperature did not increase above 38.5°C (Formenti *et al.*, 1992). This clinical study demonstrates that local temperatures in the peritoneal cavity can be raised while systemic temperatures show only a minor increase. Recently we demonstrated in the same model that the increase in platinum concentration in normal tissues after the combined treatment remained behind of that in tumours. Except in the kidney, in the case of cDDP treatment combined with hyperthermia, no other severe increase in tissue platinum concentration resulting in toxicity could be observed (Los *et al.*, 1991*b*, 1992).

At elevated temperatures the structure of biomembranes can be altered drastically since membrane lipids and proteins are in dynamic equilibrium with each other. The physical state of the lipid component of the membrane may have significant effects on the properties of the proteins and as such modulate their conformation and activity (Konings *et al.*, 1988). Increasing the temperature may result in an increased permeability of the cell membrane (Arancia, 1989). This may partly explain the increased platinum concentrations in peritoneal tumours after i.p. cDDP or CBDCA treatment combined with regional hyperthermia of the peritoneal cavity in this study. Since the pharmacokinetic profiles of cDDP and CBDCA are also altered, it is not excluded that an increase in tumour exposure also contributes to the higher intratumoral platinum concentrations. However, *in vitro* studies with CC531 carcinoma cells have also demonstrated increased intracellular platinum concentrations after incubation with cDDP or CBDCA at higher temperatures, supporting the increased permeability hypothesis (Los *et al.*, 1991*b*). In addition, previous *in vitro* work has demonstrated that temperatures in the range of 37–41.5°C do not affect the binding of either cDDP or CBDCA to isolated salmon sperm DNA in solution (Los *et al.*, 1993*b*), indicating that the binding *per se* of cDDP and CBDCA to DNA is not influenced by increased temperatures. Therefore, it is likely that the increased platinum concentrations found in peritoneal tumour after hyperthermia treatment resulted in higher levels of cDDP and CBDCA DNA adducts. cDDPDNA adducts were also detectable after cDDP treatment at 37°C, although the staining density for the CBDCA DNA adducts did not exceed the detection limit of this immunocytochemical method after normothermic treatment. This difference is probably due to the lower hydration rate of CBDCA compared with cDDP. It is known that the hydration rate of CBDCA can be as much as 100 times lower than that of cDDP (Knox *et al.*, 1986), resulting in a lower DNA binding for the same molar exposure concentration.

As a result of the higher intratumoral platinum concentrations and the increased platinum DNA adducts, chemo-hyperthermia treatment showed an enhanced anti-tumour response compared with chemotherapy alone. At two different sites in the peritoneal cavity, namely on the peritoneal wall and on adipose tissue surrounding the spermatic cord located high in the pelvis, increased tumour growth delay was shown after raising the temperature. Comparing the tumour growth delay induced by cDDP or CBDCA in combination with abdominal hyperthermia, one may conclude that the tumour growth delay after cDDP treatment increased with a small increase in temperature, whereas higher temperatures were required for CDBCA (Table II). These findings in the animal model corresponded with the *in vitro* observations. Cytotoxicity after CBDCA treatment ($IC_{50}$ dose) increased significantly at 41.5°C and above, while cytotoxicity of cDDP ($IC_{40}$ dose) increased at 38.5°C, indicating different degrees of potentiation for cDDP and CBDCA at an $IC_{40/50}$ dose. In an additional experiment at 42°C (data not shown), potentiation of CBDCA reached the same level as cDDP at 41.5°C. This confirmed the suggestion that CBDCA needs higher temperatures than cDDP for the potentiation of its cytotoxic effect.

For i.p. therapy to show an advantage over i.v. therapy, the drug is required to diffuse inward from the periphery into the intraperitoneal tumour mass. By combinding i.p. cDDP or CBDCA chemotherapy with abdominal hyperthermia one would expect a better penetration capacity of the cytostatic drug. We hypothesise therefore that one of the factors involved in the increase in intratumoral platinum concentration, resulting in an increase in tumour growth delay for both cDDP and CBDCA, is better penetration of cDDP and CBDCA into tumour tissue. This hypothesis is partly supported by previous work in which the spatial platinum distribution in peritoneal tumours after i.p. cDDP treatment at 41.5°C was more homogeneous than after treatment at 37°C (Los et al., 1991b). However, such an effect was not demonstrated for CBDCA. Previous work has demonstrated that abdominal hyperthermia augments intratumoral platinum concentrations regardless of whether CBDCA is delivered i.p. or i.v. (Los et al., 1992). Nevertheless, a rationale for pursuing the i.p. route for CBDCA was given by concerns over possible enhanced nephrotoxicity. It was demonstrated that Pt concentrations in the kidney are increased more after i.v. chemo-hyperthermia treatment than after i.p. chemo-hyperthermia (Los et al., 1992).

In addition, other groups have now shown that hyperthermia increases the cytotoxicity of several chemotherapeutic agents, such as nitrogen mustard, cisplatin (cDDP), carboplatin (CBDCA), bleomycin and mitomycin (Hahn, 1979; Barlogie et al., 1980; Dahl, 1988). Fujimoto et al. (1988) observed in an *in vivo* model of gastric cancer xenotransplanted into nude mice that hyperthermia plus mitomycin C led to a delay in tumour growth and a significantly greater effect on DNA synthesis than hyperthermia alone. The same group also demonstrated that gastric cancer patients with peritoneal dissemination treated with an intraperitoneal hyperthermic perfusion with mitomycin C had a longer disease-free survival period than after systemic mitomycin C alone (Kokubun et al., 1991). Taking into account the relative magnitude of the effects on tumours and normal tissues, no tissues other than the kidney seem to be negatively affected. Previous findings together with those presented in the present study suggest that the combination of chemotherapy and hyperthermia may be beneficial.

We would like to thank Dr Leo den Engelse for providing antibody NKI-A59 and Martin van der Vlist for excellent technical assistance. The work was supported by Grant NKI 90-19 from the Dutch Cancer Society (Koningin Wilhelmina Fonds).

### References

ALBERTS, D.S., YOUNG, L., MASON, N. & SALMON, S.E. (1985). *In vitro* evaluation of anticancer drugs against ovarian cancer at concentrations achievable by intraperitoneal administration. *Semin. Oncol.*, **12(3)**, 38–42.

ARANCIA, G., CRATERI TROVALUSCI, P., MARIUTTI, G. & MONDOVI, B. (1989). Ultrastructural changes induced by hyperthermia in Chinese hamster V79 fibroblasts. *Int. J. Hyperthermia*, **5**, 341–350.

BARLOGIE, B., CORRY, P.M. & DREWINKO, B. (1980). *In vitro* hyperthermochemotherapy of human colon cancer cells with cisdichlorodiammineplatinum(II) and mitomycin C. *Cancer Res.*, **40**, 1165–1168.

BEGG, A.C. (1987). Principles and practice of the tumor growth delay assay. In *Rodent Tumor Models in Experimental Cancer Therapy*, Kallman R.F. (ed.) pp. 114–121. Pergamon Press: Oxford.

DAHL, O. (1988). Interaction of hyperthermia and chemotherapy. *Recent Results in Cancer Res.*, **107**, 157–169.

DUNNICK, N.R., JONES, R.B., DOPPMAN, J.L. SPEYER, Y. & MYERS, E. (1979). Intraperitoneal contrast infusion for assessment of intraperitoneal fluid dynamics. *Am. J. Roentgenol.*, **133**, 221–223.

ELFERINK, F., VAN DER VIJGH, W.J.F., KLEIN, I., TEN BOKKEL HUININK, W.W., DUBBELMAN, R. & MCVIE, J.G. (1988). Pharmacokinetics of carboplatin after intraperitoneal administration. *Cancer Chemother. Pharmacol.*, **21**, 57–60.

FISHER, G.A. & HAHN, G.M. (1982). Enhancement of cis-platinum(II)diammine-dichloride cytotoxicity by hyperthermia. *Natl Cancer Inst. Monogr.*, **61**, 255–257.

FORMENTI, S., SAPOZINK, M.D., MORROW, P., SCHLAERTH, J., GABOR, J., JEFFERS, J., CHAN, K. & MUGGIA, F. (1992). Regional hyperthermia (HT) and carboplatin (CB) for ovarian cancer (OC), initial thermometry, clinical and pharmologic results. *Proc. Am. Soc. Clin. Oncol.*, **11**, 238.

FUJIMOTO, S., OHTA, M., SHRESTHA, R.D., KOKUBIN, M., MIYOSHI, T., MORI, T., ARIZUMI, N. & OKUI, K. (1988). Enhancement of hyperthermochemotherapy for human gastric cancer in nude mice by thermosensitization with nitroinidazoles. *Br. J. Cancer*, **58**, 42 45.

HAHN, G.M. (1979). Potential for therapy of drugs and hyperthermia. *Cancer Res.*, **39**, 2264–2268.

HAHN, G.M. & SHIU, E.C. (1983). Effect of pH and elevated temperatures on the cytotoxicity of some therapeutic agents on Chinese hamster cells *in vitro*. *Cancer Res.*, **43**, 5789–5791.

HAHN, G.M., BRAUN, J. & HAR-KEDAR, I. (1975). Thermochemotherapy: synergism between hyperthermia (42–43°C) and adriamycin (or bleomycin) in mammalian cell inactivation. *Proc. Natl Acad. Sci. USA*, **172**, 937–940.

HERMAN, T.S., TEICHER, B.A., CHAN, V., COLLINS, L.S., KAUFMAN, M.E. & LOH, C. (1988). Effect of hyperthermia on the action of cis-diammine-dichloroplatinum (II), rhodamine-$123_2$-[tetrechloroplatinum (II)], rhodamine 123, and potassium tetrachloroplatinum (II) *in vitro* and *in vivo*. *Cancer Res.*, **48**, 2335–2341.

HOWELL, S.B., PFEIFLE, C.E., WUNG, W.E., OLHSEN, R.A., LUCAS, W.E., YON, J.L. & GREEN, M. (1982). Intraperitoneal cisplatin with systemic thiosulfate protection. *Ann. Intern. Med.*, **97**, 845–851.

KERR, D.J. & KAYE, S.B. (1987). Aspects of cytotoxic drug penetration with particular reference to anthracycylines. *Cancer Chem. Pharmacol.*, **19**, 1–5.

KNOX, R.J., FRIEDLOS, F., LYDALL, D.A. & ROBERTS, J.J. (1986). Mechanism of cytotoxicity of anticancer platinum drugs: evidence that cis-diamminedichloroplatinum(II) and cis-diammine-(1,1-cyclo-butanedicarbocylato)platinum(II) differ only in the kinetics of their interaction with DNA. *Cancer Res.*, **46**, 1972–1979.

KOKOBUN, M., FUJIMOTO, S., SHRESTHA, R.D., KOBAYASHI, K., KIUCHI, S., KONNO, C., TAKAHASHI, M., OHTA, M. & OKUI, K. (1991). Intraperitoneal hyperthermic perfusion treatment for patients with gastric cancer and peritoneal implantation. *Reg. Cancer Treat.*, **3**, 316–319.

KONINGS, A.W.T. (1988). Membranes as targets for hyperthermic cell killing. *Recent Results in Cancer Research*, **109**, 9–21.

LEVIN, L. & HRYNIUK, W.M. (1987). Dose intensity analysis of chemotherapy regimens in ovarian carcinoma. *J. Clin. Oncol.*, **5**, 756–767.

LOS, G., MUTSAERS, P.H.A., VAN DER VIJGH, W.J., BALDEW, G.S., DE GRAFF, P.W. & MCVIE, J.G. (1989). Direct diffusion of cis-diamminedichloroplatinum (II) in intraperitoneal rat tumors after intraperitonal chemotherapy: A comparison with systemic. *Cancer Res.*, **49**, 3380–3384.

LOS, G., MUTSAERS, P.H.A., LENGLET, W.J.M., BALDEW, G.S. & MCVIE, J.G. (1990). Platinum distribution in intraperitoneal tumors after intraperitoneal cisplatin treatment. *Cancer Chemother. Pharmacol.*, **25**, 389–394.

LOS, G. & MCVIE, J.G. (1991). Experimental and clinical status of intraperitoneal chemotherapy. *Eur. J. Cancer*, **26**, 755–762.

LOS, G., VERDEGAAL, E.M.E., MUTSAERS, P.H.A. & MCVIE, J.G. (1991a). Penetration of carboplatin and cisplatin into rat peritoneal tumor nodules after intraperitoneal chemotherapy. *Cancer Chemother. Pharmacol.*, **28**, 159–165.

LOS, G., SMINIA, P., WODERGEM, J., MUTSAERS, P.H.A., HAVEMAN, J., TEN BOKKEL HUININK, D., SMALS, O., GONZALEZ GONZALEZ, D. & MCVIE, J.G. (1991b). Optimisation of intraperitoneal cisplatin therapy with regional hyperthermia in rats. *Eur. J. Cancer*, **27**, 472–477.

LOS, G., SMALS, O.A.G., VAN VUGT, M.J.H., VAN DER VLIST, M., DEN ENGELSE, L., MCVIE, J.G. & PINEDO, H.M. (1992). A rationale for carboplatin treatment and abdominal hyperthermia in cancers restricted to the peritoneal cavity. *Cancer Res.*, **52**, 1252–1258.

LOS, G., TUYT, L., VAN VUGT, M.J.H., SCHORNAGEL, J. & PINDEO, H.M. (1993a). Combination treatment of cis- and carboplatin in cancers restricted to the peritoneal cavity of the rat. *Cancer Chemother. Pharmacol.*, **32**, 425–433.

LOS, G., VAN VUGT, M.J.H., DEN ENGELSE, L. & PINEDO, H.M. (1993b). Effect of temperature on the interaction of cisplatin and carboplatin with cellular DNA. *Biochem. Pharmacol.* (in press).

MARKMAN, M. (1991). Intraperitoneal chemotherapy. *Sem. Oncol.*, **18**, 248–254.

MARQUET, R.L., WESTBROEK, D.J. & JEEKEL, J. (1984). Interferon treatment of transplantable rat colon adenocarinoma: importance of tumor site. *Int. J. Cancer*, **33**, 689–692.

OZOLS, R.F., LOCKER, G.Y. & DOROSHOW, J.H. (1979). Pharmacokinetics of adriamycin and tissue penetration in murine ovarian carcinoma. *Cancer Res.*, **39**, 3209–3213.

RIVIERE, J.E., PAGE, R.L., DEWHIRST, M.W., TYCKOWSKA, K. & THRALL, D.E. (1986). Effect of hyperthermia on cisplatin pharmacokinetics in normal dogs. *Int. J. Hypertherm.*, **2**, 351–358.

SCHERER, E., VAN BENTHEM, J., TERHEGGEN, P.M.A.B., VERMEULEN, E., WINTERWERP, H.H.K. & DEN ENGELSE, L. (1988). Immunocytochemical analysis of DNA adducts at the single cell level: a new tool for experimental carcinogenesis, chemotherapy and molecular epidemiology. Bartsch, H., Hemminki, K. & O.Neil, I.K. (eds). In: *Detecting Methods for DNA Damaging Agents in Humans: Applications in Cancer Epidemiology and Prevention*, IARC:Lyon. pp. 286–281. IARC scientific publication no. 89.

SPRATT, J.S., ADCOCK, R.A. & SHERRILL, W. (1980). Hyperthermic peritoneal perfusion systems in canines. *Cancer Res.*, **40**, 253–255.

TEN BOKKEL HUININK, W.W., DUBBELMAN, R., AARTSEN, A., FRANKLIN, H. & MCVIE, J.G. (1985). Experimental and clinical results with intraperitoneal cisplatin. *Semin. Oncol.*, **12**, 43–46.

TERHEGGEN, P.M.A.B., FLOOT, B.G.J., SCHERER, E., BEGG, A.C., FICHTINGER-SCHEPMAN, A.M.J. & DEN ENGELS, L. (1987). Immunocytochemical detection of interaction products of cisdiammine-dichloroplatinum (II) and cis-diammine(1,1-cyclobutane dicarboxylato)platinum(II) with DNA in rodent tissue sections. *Cancer Res.*, **47**, 6719–6726.

TERHEGGEN, P.M.A.B., FLOOT, B.G.J., LEMPERS, E.L.M., VAN TELLINGEN, O., BEGG, A.C. & DEN ENGELSE, L. (1991). Antibodies against cisplatin-modified DNA and cisplatin-modified (di)nucleotides *Cancer Chemother. Pharmacol.*, **28**, 185–191.

WEST, G.W., WEICHSELBAUM, R. & LITTLE, J.B. (1980). Limited penetration of methotrexate into human osteosarcoma spheroids as a proposed model for solid tumour resistance to adjuvant chemotherapy. *Cancer Res.*, **40**, 3665–3668.

WONDERGEM, J., BULGER, R.E., STEBEL, F.R., NEWMAN, R.A., TRAVIS, E.L., STEPHANS, C.L. & BULL, J.M.C. (1988). Effect of cisdiamminedichloroplatinum(ii) combined with whole body hyperthermia on renal injury. *Cancer Res.*, **48**, 440–446.

ZAKRIS, E.L., DEWHIRST, M.W., RIVIERE, J.E., HOOPES, P.J., PAGE, R.L. & OLESON, J.R. (1987). Pharmokinetics and toxicity of intraperitoneal cisplatin combined with regional hyperthermia. *J. Clin. Oncol.*, **5**, 1613–1620.